# Relationship Between Body Mass Index and Medical Care Expenditures for North Carolina Adolescents Enrolled in Medicaid in 2004

**Published:** 2007-12-15

**Authors:** Paul A. Buescher, J. Timothy Whitmire, Marcus Plescia

**Affiliations:** State Center for Health Statistics; North Carolina Division of Public Health, Raleigh, North Carolina; North Carolina Division of Public Health, Raleigh, North Carolina

## Abstract

**Introduction:**

Many studies document that overweight and obese adults have substantially higher medical care expenditures than do adults of normal weight, but comparable data for children or adolescents are few. This study examines patterns of expenditure for medical care and use of medical care services among a sample of North Carolina adolescents enrolled in Medicaid, stratified by body mass index categories.

**Methods:**

North Carolina public health records, which include clinically measured height and weight, were linked to 2004 North Carolina Medicaid enrollment records to find adolescents aged 12–18 years whose records matched. We then examined all paid claims for 2004 of the 3528 adolescents whose records matched. Total expenditures by sex and race, hospital costs, physician costs, and prescription drug costs were tabulated and stratified by body mass index. We also examined, by body mass index, the percentage of adolescents who had a paid claim for selected diagnosed health conditions.

**Results:**

Overall, and for most demographic and service categories, overweight adolescents and at-risk-for-overweight adolescents had higher average Medicaid expenditures than did normal-weight adolescents. Some of these differences were statistically significant. Overweight adolescents were significantly more likely to have a paid claim for services related to diabetes, asthma, or other respiratory conditions.

**Conclusion:**

Although based on a small sample, our results suggest that overweight has negative health consequences as early as adolescence. Further studies with larger samples could help confirm the findings of our study.

## Introduction

Obesity is now recognized as a serious public health epidemic and a significant underlying cause of morbidity and mortality in the United States (1,2). The prevalence of overweight and obesity has grown dramatically during the last decade: two-thirds of adults in the United States are overweight or obese (3). The largest increases in overweight and obesity are among children and people of minority races (3). Recent U.S. data show that 17% of children and adolescents aged 2–19 years are overweight and that another 17% are at risk for overweight (3). Overweight children are more likely than normal-weight children to be obese as adults (4), and being overweight during childhood increases the risk for adult cardiovascular morbidity (5). Overweight during childhood also has immediate consequences, including psychosocial problems, hypertension, high cholesterol, and abnormal glucose tolerance (6).

Adults who are overweight or obese have substantial medical care expenses: in 1998, the direct medical costs of obesity accounted for 9.1% of all U.S. medical expenditures. Medicare and Medicaid pay about half of these costs (7). Among the U.S. states, the obesity-attributable expenses of the Medicaid system in 2003 ranged from $23 million in Wyoming to $3.5 billion in New York (8). Most studies of the direct costs of obesity focus only on adults and use statistical modeling methods to estimate the costs for all U.S. adults and for specific populations such as Medicaid beneficiaries. Several studies examined the costs and patterns of use associated with university health care systems or large health maintenance organizations (9-11). These studies show that health care costs are significantly higher for obese adults than for adults of normal weight. However, few data document health care use and costs related to overweight children.

The purpose of our study was to analyze patterns of health care use and related costs to the Medicaid program for overweight adolescents who seek health care in public health clinics.

## Methods

We used Medicaid's paid claims and enrollment records to identify patterns of health care use and to determine whether medical costs vary significantly among groups of North Carolina adolescents classified by body mass index (BMI). To determine BMIs, we linked the Medicaid data with the data of the North Carolina Nutrition and Physical Activity Surveillance System (NC–NPASS), which include data on height and weight of children who seek health care at public health clinics, some school health centers, and at clinics run by the Special Supplemental Nutrition Program for Women, Infants, and Children (WIC). Height and weight are measured at these clinics by health professionals. We selected adolescents aged 12–18 years for our study, on the assumption that any association between BMI and medical care expenditures would be greater for this age group than for younger age groups. The NC–NPASS data system had records for 7628 adolescents aged 12–18.

About 80% of children in the NC–NPASS are aged 2–4; many are in the system because they participate in WIC. However, since eligibility for WIC ends at age 5, no WIC participants were eligible for our study. About 58% of 12- to 18-year-olds who seek care in North Carolina public health clinics have their height and weight recorded and are therefore in the NC–NPASS system.  The other 42% either did not have their height and weight measured while in the clinic or their height and weight were measured but not reported to the health department patient information system that is the source for the NC–NPASS data.

We linked the NC–NPASS records to the Medicaid enrollment records using several iterations of name, date of birth, county of residence, and Medicaid identification (ID) number. In the first iteration, matching was done by last name, first name, date of birth, and county of residence (2878 matches). Nonmatches from this step were then linked by the first five letters of the last name, first three letters of the first name, and date of birth (544 matches). Records not matching in the first two iterations were finally linked on Medicaid ID number (106 matches). Medicaid records were included in the matching process only if the adolescent was enrolled for at least 11 months during 2004 because the medical care expenditure records of anyone enrolled in Medicaid for fewer than 11 months may not be complete. Of the 7628 NC–NPASS records for 12- to 18-year-olds, 3528 matched with the Medicaid files. Some records did not match because the variables did not match perfectly. However, most nonmatching occurred because many 12- to 18-year-olds in the NC–NPASS system received health care at public health clinics but were not enrolled in Medicaid at all or were not enrolled for at least 11 months during 2004. 

The number of adolescents eligible for our study was 3528. We compared the BMI distribution of those 3528 adolescents with the BMI distribution of all 7628 adolescents aged 12–18 in NC–NPASS to see how well our matched study sample represented all adolescents aged 12–18 in NC–NPASS. We also compared our group of 3528 adolescents with all 12- to 18-year-olds enrolled in Medicaid in 2004 for at least 11 months by age, sex, and race to see how well our sample represented the total adolescent Medicaid population.

For each of the 3528 adolescents aged 12–18, we used the Medicaid ID number to extract the data on all their claims paid by Medicaid during 2004. We then tabulated the average total Medicaid expenditure per child for the following BMI categories: overweight (95th or higher percentile), at risk of overweight (85th to 94th percentile), normal weight (5th to 84th percentile), and underweight (lower than the 5th percentile). The percentiles were based on the Centers for Disease Control and Prevention 2000 BMI Reference (12). We also tabulated these results by race and sex.

We divided the expenditure results into several categories: hospital costs, physician costs, and prescription drug costs. We also calculated, by BMI, the percentage of adolescents who had one or more paid claims for selected diagnosed health conditions.

To compute average expenditures, we used total expenditures for all adolescents in the weight group as the numerator and the total number of adolescents in the weight group as the denominator. Some adolescents did not have any claims during the year, but they were still included in the denominator, since our aim was to show average expenditures per adolescent in the group, not just per adolescent who made a claim.  Many adolescents had no claims during the year for hospital costs or for some of the selected health conditions. For example, only 4% of the adolescents in our sample were hospitalized during 2004.

We used difference of means tests to see which expenditure differences by BMI category were statistically significant at the 95% confidence level, using those with normal weight as the reference group. We used chi square tests with Yates's correction to test for differences between the percentage of adolescents with each selected health condition, comparing adolescents in the normal-weight group with those in each of the other BMI groups. We used the conservative two-tail statistical tests, and we emphasize that this descriptive study shows only associations between BMI and medical care costs, not cause and effect.

## Results

In 2004, 184,523 adolescents aged 12–18 were enrolled in Medicaid for at least 11 months. Table 1 compares our sample of 3528 with the 184,523 Medicaid enrollees. We found that our sample was younger: 44.7% were aged 12 or 13 versus 34.5% in the total Medicaid group. Conversely, 12.7% of adolescents in our sample were aged 17 or 18, compared with 20.9% in the total Medicaid group. The percentage distributions of the two groups by sex and race were similar, except that the Medicaid group had a higher percentage in the "other races" category.

Table 2 shows the distribution by BMI category of all adolescents aged 12–18 in the 2004 NC–NPASS database, compared with the BMI distribution of our sample of 3528. The weight distribution of all adolescents who used public health clinics in North Carolina and who had their height and weight measured is similar to the distribution for our matched sample: 27.2% of the total NC–NPASS group were overweight, and 28.9% of our sample were overweight.

Table 3 shows the number and percentage of adolescents in our study sample and the average Medicaid expenditure per adolescent by sex, race, and weight category. Overall, 28.9% of the adolescents in our sample were overweight, 17.5% were at risk for overweight, 52.2% were normal weight, and 1.4% were underweight. These percentages varied little by sex or race. 

Average expenditures during 2004 were consistently higher for the overweight and the at-risk-for-overweight groups than for the normal-weight group (Table 3). Overall, the at-risk-for-overweight group had Medicaid expenditures that were 33% higher than those for the normal-weight group, and the overweight group had expenditures that were 25% higher. Average Medicaid expenditures were highest for the underweight group. An exception to this general pattern is that average expenditures for boys were higher for the normal-weight group than for the at-risk-for-overweight group, but Medicaid expenditures for overweight boys were 19% higher than for normal-weight boys. Average Medicaid expenditures for African American adolescents were 27% lower than those for white adolescents ($3609 vs $4963). The average expenditures for adolescent girls at risk for overweight were 66% higher than the average expenditures for normal-weight girls, a statistically significant difference (*P* = .02).

Table 4 shows the average expenditures by BMI category and major type of health service (hospital stay, physician visit, and prescription drug): Average Medicaid expenditures for normal-weight adolescents were the lowest, and expenditures for underweight adolescents were by far the highest. Average expenditures for visits to a physician ($2154) were 52% of the average for all Medicaid expenditures ($4113: Table 3). Average expenditures for prescription drugs for overweight adolescents were 42% higher than those for normal-weight adolescents, a statistically significant difference (*P *= .01).

Table 5 shows the average expenditures for adolescents with selected health conditions and the percentage of adolescents with each condition in each weight category who had one or more Medicaid paid claims. A significantly higher percentage of overweight adolescents had a claim for diabetes, asthma, or other respiratory conditions than did the normal-weight group (*P *<.05). A significantly lower percentage of overweight adolescents had a claim for injury *(P* = .04). As expected, overweight adolescents were much more likely to have a medical claim paid by Medicaid for which the primary diagnosis was obesity (ICD-9-CM code 278) (*P* < .001). In addition, overweight adolescents were significantly less likely than normal-weight adolescents to have had a well-child care visit during the year (*P* < .001).

As can be seen from data in Table 5, the highest average expenditure by far for any health condition was for mental disorders: expenditures for services for mental disorders were 64% of all Medicaid expenditures for this sample of adolescents ($2643 of $4113). By contrast, only 25% of Medicaid expenditures for all age groups were for mental disorders. The percentage of adolescents with one or more claims for a mental disorder did not differ much by weight category (Table 5). However, the average expenditure per adolescent who had a claim for mental disorder services was $9890 for those of normal weight, $14,734 for those at risk for overweight, and $14,810 for those who were overweight (data not shown in Table 5).

## Discussion

Children in North Carolina who receive health services from public health clinics are more likely to be overweight than are children in a representative national sample (3). Of  the 12- to 18-year-olds who had their height and weight recorded in North Carolina public health clinics in 2004, 27.2% were overweight (Table 2). The comparable figure from the 2003−2004 national sample is 17% (3). A statewide representative telephone survey of parents of North Carolina children (Child Health Assessment and Monitoring Program [CHAMP]) showed that (according to weight and height reported by a parent or guardian) 15% of 12- to 17-year-olds were overweight in 2005.  A statewide assessment of all Arkansas public school students showed that 21% of K−12 students were overweight in 2003−2004 and 2004−2005 (13). The differences between the findings of these studies and our study indicate that the children in our sample are a high-risk group.

Studies by Stettler et al (14), Gauther et al (15), and Mirza et al (16) of children who sought medical care at community health centers or inner-city clinics show percentages of overweight more similar to ours. Children who use public health clinics and community health centers are at much higher risk for obesity than are children who visit private practitioners. Like community health centers, public health clinics play an important role in health care delivery because of the populations they serve (predominantly underserved people, people of low socioeconomic status, and people from minority races). Children aged 6 years or older in North Carolina qualify for public health services only if their family income is lower than 100% of the federal poverty level. These health care clinics are an important source of care for children who are overweight and should be a focus of programs to reduce childhood obesity.

Primary caregivers' advice to patients about eating healthfully, engaging in physical activity, and viewing less television is an important intervention to prevent or reduce obesity (14,17-19). Public health clinics can be an important health care resource for controlling overweight among high-risk children. Indeed, public health clinics may give more comprehensive well-child care than do other providers. For example, a study of lead screening in North Carolina showed that public health clinics screened a higher percentage of Medicaid children for lead poisoning during check-up visits than did other providers (20).

Our study shows that a significantly lower percentage of the overweight adolescents in the sample than normal-weight adolescents had a well-child care visit. This discrepancy could be partly because overweight adolescents are more likely than normal-weight adolescents to have their height and weight reported to NC–NPASS as the result of a treatment visit. In our matched sample of 3528 adolescents, 26% of the visits by the overweight group to public health clinics were for treatment and 74% were for preventive or diagnostic services; for the normal-weight group the comparable percentages were 21 and 79. This issue needs to be explored further. In any case, efforts should be made to ensure that adequate screening and counseling services are provided to low-income adolescents who are overweight.

Overweight and obesity are risk factors and predisposing conditions for diabetes (21). Our data suggest that diabetes may develop earlier in overweight adolescents than in normal-weight adolescents. Our study also shows a significantly higher percentage of overweight adolescents than normal-weight adolescents with medical care claims for asthma and other respiratory conditions. A study by Gennuso et al (22) of urban, mostly Hispanic children and adolescents showed an association between asthma and obesity and suggested that asthma is a risk factor for obesity.  

The high average expenditures for the underweight adolescents may to some extent be due to an illness leading to weight loss. The number of underweight adolescents in our study is small, so the results should be considered with caution. Because of the small size of the underweight group, only one difference was nearly statistically significant: underweight adolescents were more likely than normal-weight adolescents to have a claim paid by Medicaid with a diagnosis in the "ill-defined conditions" category.

Our data show that despite a similar prevalence of overweight among African American and white adolescents, the average Medicaid expenditure was much lower for African Americans than for whites. This finding is consistent with the results of a North Carolina study of children aged 0–4 (23), which found that Medicaid expenditures for African American children were significantly lower than those for white children, even after statistically controlling for other maternal and infant characteristics that affect health service use. The researchers concluded that the unmeasured "factors contributing to the lower use of health care services among African American children might include family transportation problems, shortages in the community of health care providers who accept Medicaid patients, lack of culturally appropriate health services, barriers to service accessibility in medical offices, and racial discrimination among health care providers" (23).

We found that 64% of Medicaid expenditures for the adolescents in our study were for treating mental disorders. And our study sample is not unusual in this regard: 65% of expenditures for all 12- to 18-year-olds enrolled in Medicaid in North Carolina for at least 11 months during 2004 (N = 184,523) were for treating mental disorders. A recent national study (24) indicated that the annual number of children prescribed antipsychotic drugs jumped fivefold from 1995 to 2002. We found higher average medical care expenditures for mental disorders among overweight and at-risk-for-overweight adolescents who had a mental disorder claim than for normal-weight adolescents who had such a claim, although the percentage of overweight or at-risk-for-overweight adolescents who had one or more claims for mental disorders was not significantly higher than the percentage of normal-weight adolescents. One study using data from the National Longitudinal Survey of Youth found that young adolescents who were obese had lower levels of self-esteem than nonobese young adolescents and that decreasing self-esteem (from age 9–10 to age 13–14) among obese adolescents was associated with higher rates of sadness, loneliness, nervousness, smoking, and alcohol consumption, compared with obese adolescents whose self-esteem did not decrease (25).

A limitation of our study is that we did not have a representative sample of low-income adolescents in North Carolina. These data are specific for adolescents who sought health care at public health clinics and who were enrolled in Medicaid. In addition, only those who had their height and weight measured and recorded in the patient information system are included. In addition, overweight adolescents may be more likely than normal-weight children to be measured for height and weight, which would bias our overweight percentages. Another limitation is that this descriptive study can show only associations between BMI and medical care expenditures, not cause and effect. Health conditions such as asthma and mental disorders may lead to weight gain among adolescents.

Overall, and in most demographic and service categories, the overweight adolescents and adolescents at risk for overweight had higher average Medicaid expenditures than did normal-weight adolescents. Some of these differences are statistically significant. These results, based on a small sample, suggest that overweight is related to negative health consequences as early as adolescence. Further studies with a larger sample size could help confirm our findings.

## Figures and Tables

**Table 1:** Comparison Between Study Sample of Adolescents and All Adolescents Aged 12 to 18 Enrolled in Medicaid, by Age, Sex, and Race, North Carolina, 2004

Adolescents Aged 12 to 18 Years	Enrolled in Medicaid in 2004^a^No. (%)	Study Adolescents^a^ No. (%)
**Age (y) **		
12	31,941 (17.3)	739 (21.0)
13	31,845 (17.3)	835 (23.7)
14	29,600 (16.0)	633 (17.9)
15	27,584 (14.9)	491 (13.9)
16	25,092 (13.6)	380 (10.8)
17	22,481 (12.2)	298 (8.4)
18	15,980 (8.7)	152 (4.3)
**Sex **		
Boys	91,910 (49.8)	1683 (47.7)
Girls	92,613 (50.2)	1845 (52.3)
**Race**		
White	72,333 (39.2)	1528 (43.3)
African American	90,785 (49.2)	1849 (52.4)
Other races	21,405 (11.6)	151 (4.3)
**Total**	184,523 (100%)	3528 (100%)

a Adolescents enrolled in Medicaid for at least 11 months in 2004.

**Table 2:** Comparison Between the Study Sample of Adolescents^a^ and All Adolescents Aged 12 to 18 in the North Carolina Nutrition and Physical Activity Surveillance System (NC–NPASS), by Weight Category,^b^ 2004

Weight Category	NC–NPASS Participants	Study Adolescents

	Number (%)	Number (%)
Underweight	117 (1.5)	49 (1.4)
Normal weight	4037 (52.9)	1841 (52.2)
At risk for overweight	1396 (18.3)	619 (17.5)
Overweight	2078 (27.2)	1019 (28.9)
**Total**	7628 (100.0)	3528 (100.0)

a Adolescents enrolled in Medicaid for at least 11 months in 2004.

b Weight categories: underweight, body mass index (BMI) less than the 5th percentile; normal weight, BMI 5th to 84th percentile; at risk for overweight, BMI 85th to 94th percentile; overweight, BMI 95th or greater percentile.

**Table 3:** Average Medicaid Expenditures for 3528 Adolescents Aged 12 to 18 Enrolled in Medicaid,^a^ by Weight Category,^b^ Sex, and Race, North Carolina, 2004

	Number of Adolescents	Weight Category (%)	Medicaid Expenditures^c^	*P* Value^d^
**Weight Category**				
Underweight	49	1.4	$6598	.25
Normal weight	1841	52.2	$3604	Ref
At risk for overweight	619	17.5	$4791	.08
Overweight	1019	28.9	4491	.13
**Sex**				
*** Boys** (total)*	1682		$4193	
Underweight	28	1.7	$7532	.26
Normal weight	946	56.2	$3904	Ref
At risk for overweight	243	14.4	$3751	.88
Overweight	465	27.6	$4661	.38
*** Girls** (total)*	1846		$4041	
Underweight	21	1.1	$5353	.61
Normal weight	895	48.5	$3294	Ref
At risk for overweight	376	20.4	$5462	.02
Overweight	554	30.0	$4347	.18
**Race^e^ **				
*** Whites** (total)*	1528		$4963	
Underweight	28	1.8	$6338	.52
Normal weight	809	52.9	$4169	Ref
At risk for overweight	263	17.2	$6314	.06
Overweight	428	28.0	$5544	.16
*** African Americans** (total)*	1850		$3609	
Underweight	19	1.0	$7536	.36
Normal weight	956	51.7	$3245	Ref
At risk for overweight	334	18.1	$3768	.53
Overweight	541	29.2	$4003	.32
**All Study Adolescents**	3528		$4113	

Ref indicates reference group.

a Adolescents enrolled in Medicaid for at least 11 months in 2004.

b Weight categories: underweight, body mass index (BMI) less than the 5th percentile; normal weight, BMI 5th to 84th percentile; at risk for overweight, BMI 85th to 94th percentile; overweight, BMI 95th or greater percentile.

c Average Medicaid expenditure per child.

d *P* values of less than .05 indicate a statistically significant difference at the 95% confidence level (two-tailed test). Average Medicaid expenditure for the underweight, at-risk-for-overweight, and overweight groups are each compared by difference-of-means tests with the average expenditure for the normal weight group.

e Data on "other races" are excluded from the table because of their low numbers. Therefore, the sum of all racial groups does not equal the number of adolescents in the study.

**Table 4:** Average Medicaid Expenditure for 3528 Adolescents Aged 12 to 18,^a^ by Weight Category^b^ and Type of Health Service Provided, North Carolina, 2004

Weight Category	Number of Adolescents	Average Cost of Health Service					

		Hospital Stay	***P *Value^c^**	Physician Visit	***P *Value^c^**	Prescription Drug	***P *Value^c^**
Underweight	49	$1201	.38	$3697	.32	$639	.15
Normal weight	1841	$269	ref	$1897	Ref	$340	ref
At risk for overweight	619	$379	.37	$2456	.17	$426	.10
Overweight	1019	$360	.44	$2361	.22	$483	.01
**All Study Adolescents**	3528	$327		$2154		$401	

Ref indicates reference group.

a Adolescents enrolled in Medicaid for at least 11 months in 2004.

b Weight categories: underweight, body mass index (BMI) less than the 5th percentile; normal weight, BMI 5th to 84th percentile; at risk for overweight, BMI 85th to 94th percentile; overweight, BMI 95th or greater percentile.

c *P* values of less than .05 indicate a statistically significant difference at the 95% confidence level (two-tail test). Average expenditures for the underweight, at-risk-for-overweight, and overweight groups are each compared with the average expenditures for the normal-weight group by difference-of-means tests.

**Table 5:** Percentage of Adolescents Aged 12 to 18^a^ (N = 3528) With Selected Health Conditions, by Weight Category,^b^ North Carolina, 2004

Health Condition	Average Expend. $	Percentage of Adolescents With Health Condition						

		Underweight (n = 49)		Normal Weight (n = 1841)	At Risk^c^ (n = 619)		Overweight (n = 1019)	

		%	***P* Value^d^**	%	%	***P* Value^d^**	%	***P* Value^d^**
Infection	17	8.2	.46	12.8	12.9	.97	13.7	.50
Neoplasm	19	0.0	.85	1.4	1.6	.79	0.9	.35
Diabetes	9	0.0	.45	0.4	0.5	.99	1.7	.001
Mental disorder	2643	26.5	.64	22.6	22.0	.77	20.1	.13
Circulatory problem	38	0.0	.83	1.4	0.3	.05	2.3	.13
Asthma	13	6.1	.97	4.9	6.6	.13	7.6	.01
Other respiratory problem	54	26.5	.97	25.3	27.9	.20	30.3	.004
Digestive problem	34	14.3	.11	7.2	7.4	.90	7.6	.76
Musculoskeletal problem	62	18.4	.96	17.6	18.4	.69	17.7	.99
Ill-defined condition	98	42.9	.05	29.1	31.1	.34	30.5	.44
Injury	130	16.3	.16	26.2	25.8	.89	22.7	.04
Obesity	3	0.0	.12	0.2	1.1	.004	12.0	<.001
Well-child care	52	53.1	.62	57.7	54.4	.17	50.6	<.001

a Adolescents enrolled in Medicaid for at least 11 months in 2004.

b Weight categories: underweight, body mass index (BMI) less than the 5th percentile; normal weight, BMI 5th to 84th percentile; at risk for overweight, BMI 85th to 94th percentile; overweight, BMI 95th or greater percentile.

c At risk for overweight.

d We used chi square tests to compare the percentage of adolescents in the normal-weight group with each percentage of adolescents in the underweight, at-risk-for-overweight, and overweight groups. *P* values of less than .05 indicate differences that are statistically significant at the 95% confidence level (two-tail test).
